# Hepatoprotective activity of the extract of *Homalium letestui* stem against paracetamol-induced liver injury

**Published:** 2017

**Authors:** Jude Efiom Okokon, Joseph Oyepata Simeon, Emem Ekpo Umoh

**Affiliations:** *Department of Pharmacology and Toxicology, Faculty of Pharmacy, University of Uyo, Uyo, Nigeria*

**Keywords:** Homalium letestui, Hepatoprotective, Antioxidant

## Abstract

**Objective:**

*H* *omalium letestui *Pellegr (Flacourtiaceae) has been traditionally used by the Ibibios of Southern Nigeria to treat stomach ulcer, malaria and other inflammatory diseases and Yorubas of western Nigeria as an antidote. This study evaluates the hepatoprotective properties of the ethanol extract of the plant stem.

**Materials and Methods:**

The hepatoprotective effect of the extract of the stem of the plant (200-600 mg/kg) was evaluated by the assay of liver function parameters, namely total and direct bilirubin, serum protein and albumin, total cholesterol, alanine aminotransaminase (ALT), aspartate aminotransaminase (AST), and alkaline phosphatase activities (ALP), antioxidant enzymes like superoxide dismutase (SOD), catalase (CAT), and glutathione peroxidase (GPx), reduced glutathione (GSH) and histopathological study of the liver. Also, GCMS analysis of n-butanol fraction of the extract was carried out.

**Results:**

Administration of the extract of the stem of the plant caused a significant (p<0.05 – 0.001) dose-dependent reduction of high levels of liver enzymes (ALT, AST and ALP), total cholesterol, direct and total bilirubin as well as elevation of serum levels of total protein, albumin and antioxidant enzymes (SOD, CAT, GPx and GSH). Histology of the liver sections from extract and silymarin-treated animals showed reductions in the pathological features compared to the paracetamol-treated animals. The chemical pathological changes were consistent with histopathological observations suggesting marked hepatoprotective effect of the extract of *H. letestui* stem*.* GCMS analysis of n-butanol fraction revealed the presence of 16 bioactive compounds.

**Conclusion:**

The results show that the extract of *H. letestui* has hepatoprotective potential which may be due to the antioxidant activity of its phytoconstituents.

## Introduction

*Homalium letestui *Pellegr (Flacourtiaceae) is a forest tree that grows up to 80–100 ft and is found in the rainforest of West Africa (Hutchinson and Daziel, 1963 ▶; Keay, 1989 ▶). The plant parts, particularly stem bark and root, have been traditionally used in form of various decoctions by the Ibibios of the Niger Delta of Nigeria to treat stomach ulcer, malaria and other inflammatory diseases as well as an aphrodisiac (Okokon et al., 2006 ▶).

Okokon et al. (2013a) ▶ reported the presence of α-terpineol, vanillin, 4-phenyl isocoumarin, 3,4,5-trimethoxy phenol, 2-coumaranone, and xanthones in the extract of *H. letestui* stem. Reports of anti-plasmodial (Okokon et al., 2006 ▶), anti-diabetic (Okokon et al., 2007 ▶), anti-inflammatory and analgesic (Okokon et al., 2013a ▶), cellular antioxidant, anti-cancer, and anti-leishmanial (Okokon et al., 2013b ▶), depressant and anticonvulsant (Okokon and Davies, 2014 ▶), antibacterial (Ita and Ngochindo, 2014a ▶), *in vitro* antioxidant activity against  2,2-diphenyl-1-picrylhydrazyl (DPPH), (Ita and Ngochindo, 2014b ▶), anti-ulcer (Amazu et al., 2015 ▶) and anti-diarrheal (Antia et al., 2015 ▶) activities of the plant have been published. In this study, we report hepatoprotective activity of this plant against paracetamol-induced liver injury in order to provide scientific basis for its use in traditional medicine.

## Materials and Methods

**Plants collection**

*H. letestui *(stem) was collected from a forest in Uruan area, Akwa Ibom State, Nigeria, in July 2014. The plant was identified and authenticated by Dr. Margaret Bassey from Department of Botany and Ecological Studies, University of Uyo, Uyo, Nigeria. A specimen (FPUU 382) was deposited at Department of Pharmacognosy and Natural Medicine Herbarium.

**Extraction**

The stem was washed and dried in the shade for 2 weeks. Dried stem was further chopped into small pieces and ground to powder. The powdered material was soaked in 50% ethanol for 72 hr. The liquid filtrate was concentrated and evaporated to dryness *in vacuo* 40C using rotary evaporator. The crude ethanol extract (100 g) was further partitioned successively into n-hexane, dichloromethane, ethyl acetate and n-butanol (1L each) to give the corresponding fractions of these solvents. The liquid filtrates were concentrated and evaporated to dryness. The dry extract and fractions were stored in a refrigerator at - 4C until use.

**Animals**

Albino wistar rats (128 – 135 g) of either sex were obtained from the University of Uyo animal house. They were maintained on standard animal pellets and water *ad libitum*. Permission and approval for animal studies were obtained from the College of Health Sciences Animal Ethics committee, University of Uyo.

**Animal treatment**

A total of 36 rats of both sexes were weighed and divided into six groups of 6 animals each and treated as follows: Group 1 consisted of normal animals that were administered with distilled water (0.2 ml/kg), Group 2 was administered with distilled water 0.2 ml/kg, while groups 3, 4 and 5 were respectively administered per oral (p.o) with 250, 500 and 750 mg/kg of *H. letestui *stem extract daily for 8 days. Group 6 was treated with silymarin (100 mg/kg) (standard drug) for the same period. Paracetamol 2 g/kg, was administered to groups 2-6 on the 8th day. Twenty-four hours after paracetamol administration, animals were sacrificed under light diethyl ether vapor. Blood were collected by cardiac puncture and used immediately.

**Hematological study**

Animals were sacrificed under diethyl ether anesthesia, then, blood samples were collected from each rat by cardiac puncture using 21 gauge (21 G) needles mounted on a 5 ml syringe into ethylene diamine tetra-acetic acid (EDTA) - coated sample bottles for analysis. Hematological parameters such as full blood count (FBC), hemoglobin, (Hb), packed cell volume (PCV), platelet concentration (PLC) and total and differential white blood cell count (WBC). These parameters were analyzed using automatic hematological system (SysmexHematology – Coagulation system, Model MO-1000 I, Trans Asia, Japan).

**Evaluation of the** **protective** **effect of** **the** **extract** **against** **paracetamol** **-induced** **liver** **injury** **on biochemical parameters** **and h** **istology of** **liver** **of rats**

 Serum was separated from the blood of each animal sacrificed and stored at -20^o^C until biochemical determinations of total protein, albumin, aspartate aminotransferase (AST), alanine aminotransferase (ALT), alkaline phosphatase (ALP), total cholesterol, total and direct bilirubin. Determinations were done spectrophotometrically using Randox analytical kits according to standard procedures of manufacturer’s protocols (Reitman and Frankel, 1957 ▶). The livers of the animals were surgically removed, weighed and a part of each was fixed in 10% formaldehyde for histological processes, while the other part was washed with ice cold 0.9% NaCl and homogenates were prepared at a ratio of 1 g of wet tissue to 9 ml of 1.25% KCl by using motor driven Teflon-pestle. The homogenates were centrifuged at 7000 rpm for 10 min at 4˚C and the supernatants were used for the assays of superoxide dismutase (SOD) (Marklund et al., 1974 ▶), catalase (CAT) (Sinha, 1972 ▶), glutathione peroxidase (GPX) (Lawrence and Burk, 1976 ▶), and reduced glutathione (GSH) (Ellman, 1959 ▶).

**Gas chromatography-Mass spectrometry analysis**

Quantitative and qualitative data were determined by GC and GC-MS, respectively. The fraction was injected onto a Shimadzu GC-17A system, equipped with an AOC-20i autosampler and a split/splitless injector. The column used was an DB-5 (Optima-5), (30 m ×0.25 mm i.d. × 0.25 µm df) coated with 5 % diphenyl-95 % polydimethylsiloxane, operated with the following oven temperature program: 50 °C, held for 1 min, rising at 3 °C/min to 250 °C, held for 5 min, rising at 2 °C/min to 280 °C, held for 3 min; injection temperature and volume, 250 °C and 1.0 µl, respectively; injection mode, split; split ratio, 30:1; carrier gas, nitrogen at 30 cm/s linear velocity and inlet pressure 99.8 KPa; detector temperature, 280 °C; hydrogen, flow rate, 50 ml/min; air flow rate, 400 ml/min; make-up (H_2_/air), flow rate, 50 ml/min; sampling rate, 40 ms. Data were acquired by means of GC solution software (Shimadzu). Agilent 6890N GC was interfaced with a VG Analytical 70-250s double-focusing mass spectrometer. Helium was used as the carrier gas. The MS operating conditions were: ionization voltage 70 eV, ion source 250 °C. The GC was fitted with a 30 m × 0.32 mm fused capillary silica column coated with DB-5. The GC operating parameters were identical with those of GC analysis described above.

Identification of components present in various active fractions of the plant extracts was based on direct comparison of the retention times and mass spectral data and those for standard compounds, and by computer matching with the Wiley and Nist Library, as well as by comparison of the fragmentation patterns of the mass spectra with those reported in the literatures (Adams, 2001 ▶; Setzer et al., 2007 ▶).

**Statistical analysis**

 Data obtained from this work were analyzed statistically using Student's t-test and ANOVA (One-way) followed by a post-test (Tukey-Kramer multiple comparison test). Differences between means were considered significant at 0.1% and 5% level of significance i.e p ≤ 0.001 and 0.05.

## Results

**Evaluation of effect of** ***Homalium letestui*** **stem on liver function test of paracetamol-induced liver injury in rats**

Administration of paracetamol (2 g/kg) to rats caused significant (p<0.001) elevation of enzymes levels such as AST, ALT, ALP, total cholesterol, total and direct bilirubin and decreased total protein and albumin levels when compared to control. Pre-treatment with the extract of *H. letestui* stem (250 – 750 mg/kg) caused significant (p<0.01-0.001) decreases of these enzymes levels and that of total cholesterol, total and direct bilirubin in the extract-treated groups when compared to the paracetamol group*.* It should be noted that the decreases were dose-dependent. Total protein and albumin levels were significantly (p<0.05-0.001) elevated is a dose-dependent manner in the groups pre-treated with the extract of the stem when compared to the paracetamol group. The effects of the highest dose of the extract on all of the parameters evaluated were comparable to those of silymarin (Table 1).

**Table 1:** Effect of *H. Letestui* on liver function of paracetamol –induced liver injury in rats

**Parameters/** **Treatment**	**Total protein** **(g/dl)**	**Albumin** **(g/dl)**	**Total bilirubin** **(mg/dl)**	**Direct bilirubin** **(mg/dl)**	**Ast** **(iu/l)**	**Alt** **(iu/l)**	**Alp** **(iu/l)**	**Total cholesterol** **Mmol/l**	**Liver weight (g)**
**Control**	6.75± 0.14	4.12±0.68	3.61±0.18	1.21±0.71	109.3±3.04	35.33±3.59	202.15±10.75	3.98± 0.41	6.53±0.23
**PCM +Dist. water**	3.43±0.27^c^	1.66±0.22^c^	5.01±0.16^c^	1.31±0.09^c^	168.3±3.46^c^	96.50±4.07^c^	303.0±9.50^c^	7.67±0.25^c^	8.46± 0.16^c^
**HL. 250mg/kg+ PCM**	6.33±0.72^e^	4.40±0.29^d^	4.85±0.18^b^	0.60±0.10^b^	147.8±3.73^ce^	43.50±3.97^f^	283.0±4.60^c^	6.70±0.19^cd^	7.63±0.20^bd^
**HL. 500mg/kg+ PCM**	6.53±0.97^e^	4.42±0.93^e^	4.95±0.29^b^	0.80±0.06^f^	129.1±5.09^af^	59.33±5.33^af^	278.5±5.14^cd^	6.54±.09^bf^	6.76±0.14^f^
**HL. 750mg/kg+ PCM**	6.39±0.74^e^	4.81±0.31^Sf^	4.11±0.09	0.73±0.09^f^	126.2±5.80^af^	75.83±3.60^ef^	276.66±5.85^cd^	5.98±0.24^f^	6.62±0.15^f^
**Silymarin 100 mg/kg + PCM**	6.72±0.11^f^	4.27±0.99^f^	4.16±0.44	1.20±0.22^f^	112.6±7.63^f^	44.16±3.48^f^	248.16±9.92^cf^	5.05±0.29^f^	6.56±0.12^f^

Data were expressed as mean±SEM. Significant at ap< 0.05, bp< 0.01 and cp< 0.001 when compared to control. Significant at dp< 0.05, ep< 0.01 and f< 0.001 when compared to paracetamol . PCM: paracetamol . n = 6.

**Effect of stem extract on liver weight**

The liver weights of rats treated with paracetamol were significantly (p<0.001) increased when compared to those of the control group. However, the weights of the animals in groups pre-treated with the extract of the stem and silymarin were significantly (p<0.01 – 0.001) reduced when compared to control (Table 1).

**Effect of stem extract on the levels of liver antioxidant enzymes**

Paracetamol treatment caused significant (p<0.001) decreases in the activities of SOD, catalase, GPx and GSH level in liver tissue when compared to control group. Pre-treatment with the extract of *H. letestui *stem (250 – 750 mg/kg) resulted in significant (p<0.05 – 0.001) increases in the activities of SOD, catalase, GPx and GSH level. Silymarin-treated animals also showed a significant (p<0.001) increase in antioxidant enzymes, namely SOD, catalase, GPx activities and GSH level compared to paracetamol-treated rats (Table 2).

**Effect of treatment with ethanol extract of** ***Homalium letestui*** **stem on hematological parameters, in rats with paracetamol-induced hepatotoxicity**

Administration of paracetamol (2 g/kg) to rats did not significantly affect (p<0.05) RBC and WBC counts as well as hemoglobin concentration, PCV and neutrophils percentages (Table 1). However, there were significant (p<0.001) reductions in the percentages of lymphocytes, monocytes and eosinophils of paracetamol-treated rats, while pretreatment with *H. letestui* stem extract caused significant (p<0.05-0.001) increases against reductions induced by paracetamol but not in a dose-dependent fashion (Table 3).

**Table 2:** Effect of *Homalium letestui* stem extract on liver antioxidant enzymes

**Parameters/** **Treatment**	**SOD** ** (U/mg of protein)**	**CAT** ** (U/mg of protein)**	**GPx** **(U/mg of protein)**	**GSH** **(µg/mg of protein)**
**Control**	22.24 ±0.24	55.52±3.26	25.38±0.66	0.35±0.01
**PCM +Dist. water**	10.32±0.12^c^	30.59± 1.15^c^	10.45±0.22^c^	0.10±0.01^c^
**HL. 250mg/kg + PCM**	14.66±0.14^d^	38.94±1.04^c^	16.74±0.19^c^	0.18±0.01^cb^
**HL. 500mg/kg+ PCM**	17.88±0.25^d^	46.32±1.15^f^	19.38 ±0.13^cf^	0.22±0.01^bf^
**HL. 750mg/kg+ PCM**	19.56± 0.11^f^	51.55±1.46^f^	20.16±0.88^bf^	0.28±0.02^af^
**Silymarin 100 mg/kg + PCM**	20.24±0.18^f^	54.01±1.94^f^	22.46±1.01^f^	0.30±0.01^f^

Data were expressed as mean±SEM. Significant at ap< 0.05, bp< 0.01 and cp< 0.001 when compared to control . Significant at dp< 0.05, ep< 0.01 and f< 0.001 when compared to paracetamol. HL:* Homalium letestui*; PCM : paracetamol. n = 6.

**Table 3:** Effect of treatment with ethanol extract of *Homalium letestui* stem on hematological parameters of rats with paracetamol-induced hepato-nephrotoxicity

**Parameters** **Treatment ** **Dose (m** **g** **/kg)**	**RBC** **(X 10** ^12^ **/l)**	**PCV** **(%)**	**Hb** **(g/dl)**	**WBC** **(X 10** ^9^ **/l)**	**Neutrophils** **(%)**	**Lymphocytes** **(%)**	**Monocytes** **(%)**	**Eosinophils** **(%)**	**Basophils** **(%)**	**Platelet**
**Control**	7.920.19	44.09.90	13.460.16	17.082.12	29.361.71	48.138.67	3.131.19	2.160.79	2.200.40	700.338.93
**PCM +** **Dist. water**	7.750.55	43.02.00	13.210.31	16.342.18	24.578.71	21.2413.32^c^	2.501.30	1.170.97^ a^	0.20.20^c^	665.315.31^c^
**HL.250 + PCM**	8.050.41	48.02.10	13.900.48	14.401.85	23.955.48	54.7011.24^ b^	2.381.30	2.501.05^c,e^	0.000.00^ c^	817.38.50^cf^
**HL.500 + PCM**	7.410.46	44.01.00	12.510.25	11.431.17	32.379.82	23.5012.05^ c^	4.620.50^c,f^	2.171.10^c,e^	0.000.00^cf^	908.598.94^cf^
**HL.750 + PCM**	8.020.28	46.01.17	13.380.40	10.321.88	20.369.05	24.1110.86^ b^	3.672.00^c,e^	2.341.17^ a^	0.000.00^cf^	640.346.10^c^
**Silymarin 100 + PCM**	7.570.20	44.21.16	13.500.35	11.661.54	16.597.88^b,f^	21.8710.07^ e^	0.500.02^ c^	1.510.70^c,e^	0.000.00^cf^	547.526.62^cf^

Data were expressed as mean±SEM. Significant at ap< 0.05, bp< 0.01 and cp< 0.001 when compared to control. Significant at dp< 0.05, ep< 0.01 and fp< 0.001 when compared to paracetamol. PCM : paracetamol; HL:* Homalium letestui*. n = 6.

**Histopathological studies of rat liver in paracetamol-induced hepatotoxicity**

**Figure 1:**
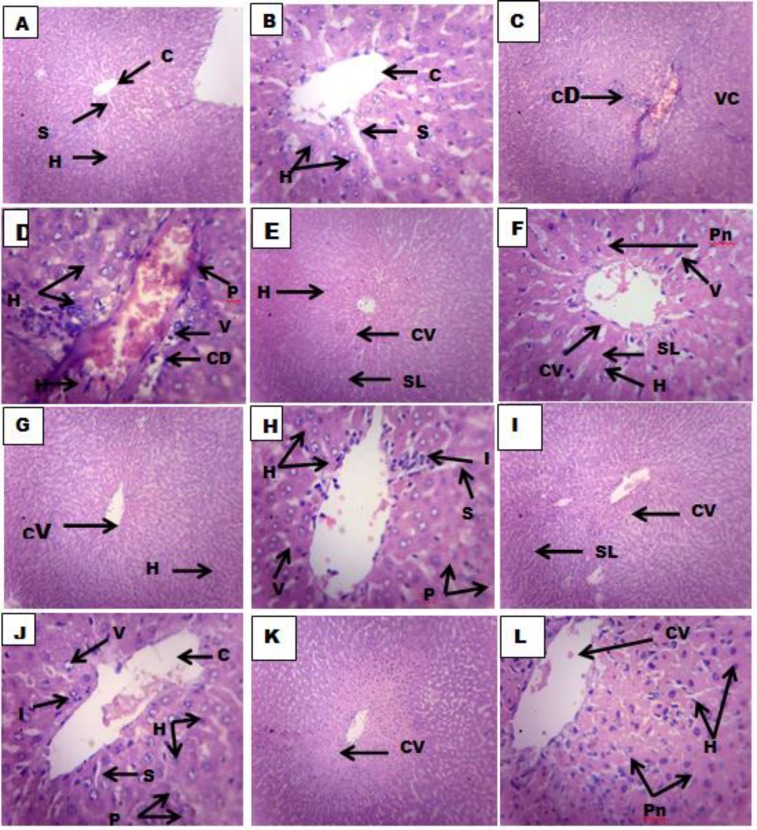
Histologic sections of the liver treated with normal saline (10 ml/kg) A (x100) and B(x400), PCM (2.0g/kg) C (x100) and D (x400), silymarin (100 mg/kg) and PCM (2.0g/kg) E (x100) and F(x400), HL (250 mg/kg) and PCM (2.0g/kg) G (x100) and H (x400), HL(500 mg/kg) and PCM (2.0g/kg) I (x100) and J (x400, HL (750 mg/kg) and PCM (2.0g/kg) K (x100) and L (x400).

Histopathological examination of liver sections of normal control group showed normal cellular architecture with distinct hepatic cells, sinusoidal spaces and central vein (Figures 1A and B). Disarrangement of normal hepatic cells with centrilobular necrosis, hyperplasia, vascular and cellular degeneration, polymorphonuclear aggregation, inflammation and fatty degeneration were observed in the paracetamol-treated rats of group 2 (Figures 1 C and D). The liver sections of the rats treated with stem extract of* H. letestui *(250 - 750 mg/kg) of groups 3, 4 and 5 showed signs of protection as shown by the reduction/ absence of inflammatory cells, vascular congestion and degeneration, cellular degeneration, necrosis and vacuoles (Figures 1 C - J), while the liver sections of the rats treated with silymarin (100 mg/kg) in group 6 showed significant reduction in fatty degeneration and absence of necrosis and inflammation (Figures 1 K and L )

**GC-MS analysis**

GCMS analysis of n-butanol fraction of *H. letestui* stem revealed the presence of 16 bioactive compounds in each fraction as represented in Table 4. 

Keys: Central vein (CV) Sinusoidal lining (SL), Hepatic Artery (HA), Hepatic vein (HV), Hepatocytes (H) and Pyknotic nucleus (Pn), Cellular degeneration (CD), Vascular congestion (VC), Vascular degeneration (VD), Hepatocytic hyperplasia (H), Sinusoidal lining (SL), Vacuolation (V), Inflammation (I), Hepatocytic hyperplasia (HH), and Cellular proliferation (CP) .

## Discussion

In the present study, the ethanol extract of *H. letestui* stem was evaluated for hepaprotective activity against paracetamol-induced hepatotoxicity in rats. Liver function test, liver antioxidant enzymes levels and histological studies were done to assess hepatoprotective properties of this plant. In the present study, paracetamol administration caused a marked depletion of tissue GSH level with reduced activities of liver antioxidative enzymes like SOD and CAT. Paracetamol caused liver toxicity as it altered various liver parameters. Also, various degrees of histological lesions were observed.

At high doses, paracetamol produces acute toxic effects which lead to liver damage. The drug is bioactivated to a toxic electrophile, *N*-acetyl *p*-benzoquinone imine (NAPQI), which covalently binds to tissue macromolecules, probably oxidizes lipids, or the critical sulfhydryl groups (protein thiols) and alters the homeostasis of calcium (Lin et al., 1997 ▶). Massive production of reactive species may lead to depletion of protective physiological moieties (glutathione and α-tocopherol, etc.), causing damage to the macromolecules in vital biomembranes and liver injury (Aldridge, 1981 ▶; Gilani et al., 2005 ▶). 

Liver function can be assessed by estimating the activities of serum ALT, AST, ALP, bilirubin (total and direct), total cholesterol, total protein and albumin which are originally present in the cytoplasm (Manokaran et al., 2008 ▶). When there is hepatopathy, these enzymes and molecules leak into the blood stream and serve as an indicator for the liver damage (Nkosi et al., 2005 ▶). Abnormally high levels of serum ALT, AST, ALP, total bilirubin (total and direct), and total cholesterol as well as decreases in total protein and albumin levels as observed in paracetamol group in our study are indications of paracetamol-induced liver dysfunction and denote the damage to the hepatic cells. The reversal of increased serum enzymes in paracetamol-induced liver damage by the extract may be due to prevention of the leakage of intracellular enzymes by its membrane stabilizing activity.

Increase in serum level of ALP is due to increased synthesis, in the presence of increasing biliary pressure (Muriel and Garcipiana, 1992 ▶) and reflects the pathological alteration in biliary flow (Plaa and Hewitt, 1989 ▶). In the present study, reduction in serum total protein and albumin levels were observed in the paracetamol-treated rats which may be associated with the decrease in the number of hepatocytes which in turn may result in decreased hepatic capacity to synthesize protein and albumin. Decreased levels of total protein and albumin as recorded in paracetamol-treated rats revealed the severity of hepatopathy. This negative effect on total protein and albumin was reversed in the groups pre-treated with the extract, indicating an improvement of the functional status of the liver cells by the extract.

Bilirubin, a metabolic product of hemoglobin, undergoes conjugation with glucuronic acid in hepatocytes to increase its water solubility. Determination of serum bilirubin represents an index for assessment of hepatic function, and any abnormal increase in the levels of serum bilirubin indicates hepatobiliary diseases and severe disturbance of hepatocellular function (Martin and Friedman, 1992 ▶). Decreased serum bilirubin level following extract treatment indicated the effectiveness of the extract in restoring normal functional status of the liver.

Paracetamol-induced toxicity in rats may have altered membrane structure and function as well as lipids metabolism in the liver as suggested by the increased cholesterol levels of rats. Alteration of bio-membrane lipid profile disturbs its fluidity, permeability, activity of associated enzymes and transport system (Cooper et al., 1977 ▶) and this could affect lipid transport in the liver. This effect was reduced by the protective activity of the stem extract which restored the level of total cholesterol to near normal.

Antioxidant enzymes are involved in scavenging superoxide anion to form hydrogen peroxide, hence reducing the toxic effect caused by these radicals. SOD and CAT are important enzymes in the enzymatic antioxidant defense system (**Curtis et al., 1972**). Decreases in their activities may result in a number of deleterious effects. In this study, it was observed that *H. letestui* stem extract significantly (p<0.05) increased hepatic SOD and CAT activities in paracetamol-induced liver damage in rats. This showed that *H. letestui* can reduce reactive free radicals, thereby reducing oxidative damage to the tissues besides improving the activity of hepatic antioxidant enzymes.

Glutathione (GSH) is one of the tripeptide and non-enzymatic biological antioxidants present in high quantities in the liver. It helps to remove free radical species such as hydrogen peroxide, superoxide radicals, alkoxy radicals and maintain membrane protein thiols, and serves as a substrate for glutathione peroxidase and glutathione transferase (Prakash et al., 2001 ▶). Reduced level of GSH is implicated in the enhancement of lipid peroxidation in paracetamol-treated rats. Pretreatment with *H. Letestui* stem extract significantly increased the level of GPx and GSH in a dose-dependent manner portraying its ability to scavenge these free radicals. 

The vital function that blood cells perform, together with the susceptibility of this highly proliferative tissue to intoxication by xenobiotics, makes the hematopoietic system a unique target organ (Dahlin et al., 1984 ▶). Certain drugs including alkylating cytotoxic agents could also affect blood formation rate and the normal range of hematological parameters (Adeneye et al., 2008 ▶). Treatment of rats with paracetamol in this study, did not significantly affect the haematological parameters such as RBC, Hb, PCV, WBC and neutrophils percentage, except for reductions in the percentages of lymphocytes, monocytes and eosinophils of paracetamol-treated rats. Pretreatment with *H. letestui* stem extract caused significant (p<0.05 -0.001) increases against reductions induced by paracetamol but not in a dose-dependent fashion. Histological findings corroborate that of the biochemical results as the degree of injury induced by paracetamol were reduced significantly by pretreatment with the stem extract.

The extract of the stem and fractions have been reported to exhibit strong cellular antioxidant activity in whole blood, neutrophils (extracellular and intracellular) and macrophages (Okokon et al., 2013b ▶) as well as *in vitro* antioxidant activity against DPPH (Ita and Ngochinda, 2014 ▶). Okokon et al. (2013a) ▶ reported the presence of α-terpineol, vanillin, 4-phenyl isocoumarin, 3,4,5-trimethoxy phenol, 2-coumaranone, and xanthones in the extract of *H. letestui* stem. Also, GCMS analysis of n-butanol fraction revealed the presence of 4-phenylisocoumarin, hexadecanoic acid, flavone, 2H-1-Benzopyran-2-one, 3-phenyl, camphene, borneol, linanool acetate, which are potential antioxidant compounds.

Vanillin, a phenolic aldehyde has been reported to possess antioxidant and free radical scavenging ability (Kamat et al., 2000 ▶; Kumar et al., 2002 ▶; Lirdprapamongkol et al., 2009 ▶) which could possibly account for the hepatoprotective property of this plant. The strong antioxidant activity of this extract explains the significant hepatoprotective activity of the stem extract. The activities of antioxidant counteract the redox state precipitated intracellularly and hence ensure hepatoprotection against paracetamol-induced liver injury. The antioxidant activity of this extract may also explain the mechanism of the hepatoprotective activity of *Homalium letestui.* The findings of this study corroborate the effect that was reported for *Homalium zeylanicum *(Shashank et al., 2011 ▶).

This study showed that *Homalium letestui* possesses strong hepatoprotective activity which is due to its antioxidant activity precipitated by its chemical constituents. This confirms the use of *H. letestui* stem as an antidote in traditional medicine.
